# Periodontal Infection Aggravates C1q-Mediated Microglial Activation and Synapse Pruning in Alzheimer’s Mice

**DOI:** 10.3389/fimmu.2022.816640

**Published:** 2022-02-01

**Authors:** Xiaoxiao Hao, Zhaofei Li, Wei Li, Jannet Katz, Suzanne M. Michalek, Scott R. Barnum, Lucas Pozzo-Miller, Takashi Saito, Takaomi C. Saido, Qin Wang, Erik D. Roberson, Ping Zhang

**Affiliations:** ^1^ Department of Pediatric Dentistry, School of Dentistry, University of Alabama at Birmingham, Birmingham, AL, United States; ^2^ Department of Neurobiology, School of Medicine, University of Alabama at Birmingham, Birmingham, AL, United States; ^3^ Department of Microbiology, School of Medicine, University of Alabama at Birmingham, Birmingham, AL, United States; ^4^ CNine Biosolutions, LLC, Birmingham, AL, United States; ^5^ Laboratory for Proteolytic Neuroscience, RIKEN Center for Brain Science, Wako, Japan; ^6^ Department of Neurocognitive Science, Institute of Brain Science, Nagoya City University Graduate School of Medical Sciences, Nagoya, Japan; ^7^ Department of Cell, Developmental and Integrative Biology, School of Medicine, University of Alabama at Birmingham, Birmingham, AL, United States; ^8^ Center for Neurodegeneration and Experimental Therapeutics, Alzheimer’s Disease Center, Department of Neurology, School of Medicine, University of Alabama at Birmingham, Birmingham, AL, United States

**Keywords:** Periodontitis, *Porphyromonas gingivalis*, Alzheimer’s disease, microglia, complement C1q, synapse loss

## Abstract

Periodontitis is a dysbiotic infectious disease that leads to the destruction of tooth supporting tissues. There is increasing evidence that periodontitis may affect the development and severity of Alzheimer’s disease (AD). However, the mechanism(s) by which periodontal infection impacts the neurodegenerative process in AD remains unclear. In the present study, using an amyloid precursor protein (APP) knock-in (*App* KI) AD mouse model, we showed that oral infection with *Porphyromonas gingivalis* (Pg), a keystone pathogen of periodontitis, worsened behavioral and cognitive impairment and accelerated amyloid beta (Aβ) accumulation in AD mice, thus unquestionably and significantly aggravating AD. We also provide new evidence that the neuroinflammatory status established by AD, is greatly complicated by periodontal infection and the consequential entry of Pg into the brain *via* Aβ-primed microglial activation, and that Pg-induced brain overactivation of complement C1q is critical for periodontitis-associated acceleration of AD progression by amplifying microglial activation, neuroinflammation, and tagging synapses for microglial engulfment. Our study renders support for the importance of periodontal infection in the innate immune regulation of AD and the possibility of targeting microbial etiology and periodontal treatment to ameliorate the clinical manifestation of AD and lower AD prevalence.

## Introduction

Alzheimer’s disease (AD), an age-related neurodegenerative disease of the central nervous system (CNS), is the most common cause of dementia and is characterized by progressive and irreversible decline in behavioral and cognitive function (1). AD affects more than 50 million people globally and is the sixth leading cause of death in the US (2). Despite great efforts, the cellular and molecular mechanisms underlying AD pathogenesis are not fully understood, and there is no effective therapy to cure or ameliorate this devastating disease (3, 4). Therefore, there is an urgent need to identify modifiable risk factors that impact AD, so that strategies that mitigate AD severity are implemented.

Periodontitis is a chronic infectious disease characterized by periodontal inflammation and alveolar bone loss, and is the primary cause of tooth loss in adults (5). Increasing evidence suggests that periodontitis is associated with a diversity of systemic disorders, including AD (6). In this regard, epidemiological studies indicate that there is a positive correlation between cognitive decline in AD patients and periodontitis (7, 8). In addition, periodontitis has been shown to positively associate with brain amyloid plaque development, a pathological hallmark of AD, in elderly and AD patients (9). Furthermore, *Porphyromonas gingivalis* (Pg), a keystone pathogen of periodontitis, as well as its virulence factors lipopolysaccharide (LPS) and gingipains, have been detected in the post-mortem brains from AD patients (10, 11). However, mechanisms underlying the association between periodontitis and the development of AD is lacking.

Microglia cells are the most common innate immune cells in the CNS and play a central role in maintaining the immune homeostasis in the brain (12). However, microglia overactivation can lead to sustained neuroinflammation and contribute to the pathogenesis of neurodegenerative diseases (13). The complement system, also known as the complement cascade, represents a major part of innate immunity and comprises an interactive network of soluble and membrane-associated proteins that activate, amplify, and regulate immunity and inflammation (14, 15). Although the complement system provides rapid recognition and response to dangers that threaten the host, a dysregulated complement system can also mediate destructive inflammation (15). Clinical and experimental studies have suggested the involvement of periodontal complement activation in the pathogenesis of periodontitis (6, 16, 17). There is also emerging evidence suggesting that aberrant complement activation is involved in AD development (18–20). However, the role complement and microglial activation in the association of periodontitis and AD is not clear.

In this study, using the amyloid precursor protein (APP) knock-in (*App* KI) mouse model, we sought to determine the effect of Pg infection on the progressive neurodegeneration of AD and the involvement of complement C1q in the process. We demonstrated the importance of micorglia and complement component C1q in the aggravation of AD neuropathologies in the presence of periodontal infection. Our study suggests a “two-hit” model of Pg-mediated aggravation of AD, with amyloid β (Aβ) accumulation as the first hit, and Pg brain invasion as the second hit to faciliate synaspse loss.

## Materials and Methods

### Mice

C57BL/6 wild-type (WT) mice were obtained from Jackson Laboratories (Ellsworth, ME, USA). *App^NL-G-F/NL-G-F^* knock-in (*App* KI) mice (21) that carry the APP Swedish (KM670/671NL), Iberian (I716F), and Arctic (E693G) mutations were originally obtained under a material transfer agreement from Dr. Takaomi Saido (RIKEN Brain Science Institute, Japan), and had been backcrossed for more than 12 generations to obtain the C57BL/6 background. Animals were housed in an environmentally controlled facility at the University of Alabama at Birmingham (UAB), protocols for all animal studies were approved by the UAB Institutional Animal Care and Use Committee.

### Bacterial Culture and Infection Model

**Figure 1:**
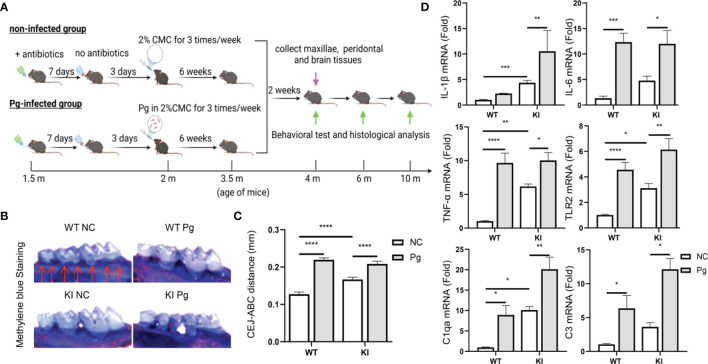
Pg-induced alveolar bone loss and periodontal inflammation in *App* KI and WT mice following oral infection. **(A)** Schematic of the experimental design used in this study. m, month. **(B)** Representative methylene blue-stained maxillae from non-infected (NC) and Pg-infected WT and *App* KI mice (n=9 mice/group). Bone loss was assessed in a total of 7 buccal sites (red arrows) per mouse. **(C)** Alveolar bone loss in Pg-infected and non-infected WT and *App* KI mice; mm, millimeter; CEJ-ABC, cemento-enamel junction-alveolar bone crest. **(D)** Inflammatory cytokine and complement gene expression in gingival tissues from non-infected and Pg-infected WT and *App* KI mice. Gene expression was normalized to GAPDH and expressed as fold changes. Samples were done in duplicate (n=7 mice/group). Data are expressed as mean ± SEM. *P < 0.05, **P < 0.01, ***P < 0.001, ****P < 0.0001, by two-way ANOVA followed with Tukey correction.

*Pg* ATCC 33277 was cultured in trypticase soy broth (BD Biosciences) containing 1% yeast extract, 5 µg/mL hemin, and 1 µg/mL menadione and grown at 37°C in an anaerobic atmosphere of 10% H2, 5% CO2, and 85% N2 (22). Pg infection was carried out as previously described (23) with some modifications (**Figure 1A**). Briefly, age- and sex-matched WT and *App* KI mice (6 weeks of age) were randomly assigned to control or Pg-infected groups. Mice were provided drinking water containing kanamycin (1 mg/ml) for 7 days to reduce the indigenous oral microflora. After removing antibiotics for 3 days, experimental mice were given 100 µl of freshly harvested Pg (10^10^ CFU/ml) in 2% carboxymethylcellulose (CMC) and control groups received 100 µl of 2% CMC by oral gavage 3 times per week for 6 weeks. Mice were refrained from food and water intake for 1 hour (h) after infection.

### Evaluation of Alveolar Bone Loss and Periodontal Inflammation

Groups of mice were sacrificed 2 weeks post the last dose of Pg to assess the establishment of periodontitis. Specifically, the right maxilla from each mouse was fixed and then stained with 1% methylene blue (24). The distance between the cementum-enamel junction (CEJ) and the alveolar bone crest (ABC) was measured at a total of seven buccal sites with the assistance of an Image J analysis system (NCBI). To assess periodontal inflammation, gingival tissues around the left maxillary molars were collected, homogenized, and analyzed for inflammatory gene expression as described below.

### Behavioral Tests

Three behavioral tests were carried out at 4, 6 and 10 months of age (**Figure 1A**). On each testing day, groups of mice were transferred to the testing room 1 h before testing for acclimatization. On day 1 of indicated age, mice were tested for general locomotor activity and exploration habits in the open field (OF) test (25). On day 2, elevated zero maze (EZM) was conducted to evaluate anxiety-related behavior (26). After resting for a day, mice were subjected to Morris water maze (MWM) for 5 days followed by probe trial to assess learning performance and reference memory, respectively (27). All behavioral tests were conducted during the light phase of the diurnal cycle and data were collected using an EthoVision video tracking system (Noduls).

### Evaluation of Aβ Peptide Accumulation in the Brain

To evaluate brain Aβ peptide accumulation, groups of mice were anesthetized and perfused with PBS (28). Brains were collected after perfusion and one side hemispheres were dissected, and cortices and hippocampi were isolated and homogenized in carbonate buffer supplemented with proteinase inhibitor cocktail (29). The supernatants were collected as the carbonate-soluble parts. The pellets were subjected to further homogenization in guanidine solution, and the supernatants were collected as the carbonate-insoluble fractions. The levels of hAβ42 in the soluble and insoluble fractions were measured using a hAβ42-specific ELISA kit (Invitrogen, #KHB3442), following the manufacturer’s instructions. The other side hemispheres were fixed in 4% paraformaldehyde (PFA) and used for immunohistochemistry staining as described below.

### Immunohistochemistry and Immunofluorescence Staining

Formalin-fixed, paraffin-embedded hemispheres were sectioned in the coronal plane at 7 µm. For analysis of Aβ plaque depositions, sections were treated with 70% formic acid after de-paraffinization and rehydration (30). Following antigen retrieval, sections were probed with an anti-Aβ antibody (6E10) (Biolegend, #803004) followed by HRP-labeled secondary antibody (JacksonImmuno, #711-035-150). A Thyramide signal amplification kit (Perkin Elmer) was used to enhance the sensitivity of detection. Nuclei were stained with DAPI. For detection of microglial and C1q activation and colocalization, sections were stained with antibodies against IBA1 (Cell signaling, #17198), and/or C1qa (Abcam, #ab182451). Data were acquired with a KEYENCE BZ-800 microscope and images were processed using an ImageJ analysis system. To examine synapse loss, mice were perfused with PBS as described above, and then further perfused with 50-60 ml 4% PFA/PBS to facilitate rapid and even fixation (31). Brain sectioned at 35 µm thickness were prepared and free-floating sections were stained for PSD95 (Cell signaling, #3450S), SNAP25 (Abcam, #ab109105), or co-stained for IBA1 or C1q. For quantitative analysis of synapses and the presence of C1q at the synapses, regions of interest (ROI) were acquired with a Zeiss LSM-800 Airyscan confocal microscope and analyzed using Puncta analyzer (Duke University) run in the ImageJ analysis system (32). About 50-100 synapses (SNAP25^+^ or PSD95^+^puncta) were recorded per mouse per ROI and the number of C1qa^+^ particles present per synapse was scored. For microglia engulfment, brain sections were imaged up to 25 Z-stacks at 0.33 µm steps (32). Ten to fifteen microglia within the hippocampus CA1 region and cortex, per mouse, were randomly selected and analyzed.

### Genomic DNA Extraction of Brain Tissue and PCR Analysis of Pg

Genomic DNA (gDNA) was extracted from mice brain tissues using a Blood and Tissue Kit (Qiagen), followed by standard PCR and qPCR to assess for Pg specific *hum*Y gene and 16S ribosomal RNA (16S rRNA), respectively (33, 34). Primer sequences are listed in **Table S1**.

### Fluorescence *In Situ* Hybridization (FISH) for Detection of Pg in Mouse Brain

FISH was conducted using probes specific to Pg 16S rRNA gene sequence on rehydrated brain sections, as previously described (35). Briefly, after deparaffinization and antigen retrieval, brain sections were probed with 5 pmol of Pg 16S rRNA-specific oligonucleotide POG (SILVA, ribosomal RNA database) 5’-CAA TAC TCG TAT CGC CCG TTA TTC-3’ labeled with fluorescein isothiocyanate (synthesized at Integrated DNA Technologies). Data were acquired with a KEYENCE BZ-800 microscope and images were processed using ImageJ.

### Quantitative Reverse Transcription PCR (RT-qPCR)

To analyze the gene expression of inflammatory cytokines and complement components in gingival tissues, brain tissues, or cultured microglia cells, tissues or cells were homogenized and total RNA were extracted using Direct-zol RNA Miniprep kit (ZYMO Research, #R2052). cDNA was synthesized using a PrimeScript RT Reagent kit (Takara, #RR037) using equal amount of RNA. RT-qPCR was performed using an ABI Prism 7500 fast system (Applied Biosystems) with the 2x qPCR Master Mix (NEB, #M3003). All primers information is listed in **Table S1**.

### Complement Component ELISA

Brain tissues were collected from groups of mice, weighed and homogenized in PBS with protease inhibitors cocktail. Homogenized brain tissues then underwent 3 freeze-thaw cycles and supernantants were collected after centrifugation. The levels of C1qa and C3 in the supernatants were assessed by ELISA according to the manufacturer’s instructions (Mybiosource, #MBS921993 and #MBS763294).

### Preparation of Oligomeric Aβ (Aβo)

Aβo was prepared as previously described (36). Briefly, the Aβ_1−42_ synthetic peptide (Bachem, #4045866) was suspended in 100% 1,1,1,3,3,3 hexafluoro‐2‐propanol (HFIP) and then incubated for complete solubilization under shaking at 37°C for 2 h. HFIP/peptide was dried in a flammable hood for 6-8 h, and re-suspended in DMSO. An oligomeric form of the peptide was further generated by pre-incubation for 24-72 h at 37°C before adding to the cultures.

### Primary Microglia Culture

Primary microglia were generated from cerebral cortices of WT newborn pups (postnatal day 0), as previously described (37). Briefly, brains were harvested, and the meninges were removed. Cortices and hippocampi were then dissected into small pieces and triturated in Hanks’ balanced salt solution (HBSS) and cultured in Dulbecco’s modified eagle medium (DMEM) containing 16 mM HEPES, 1x non-essential amino acids, 1x L-glutamine, 10% fetal bovine serum (FBS) and 50 µg/ml gentamicin for 10 days until confluent. Microglia were collected and then re-suspended in fresh media and cultured for 18-24 h prior to stimulation. Microglia (1x10^6^) were treated with Aβo (0.1 µM, 1 µM and 10 µM), Pg (MOI =50), Pg plus Aβo, or pre-treated with Aβo for 6 h followed by 24 h Pg stimulation. To test the role of complement activation in Pg-induced inflammatory responses in microglia, microglia were pre-incubated with 100 ng/ml C1 esterase inhibitor (C1-INH, Sigma, #E0518) for 4 h, followed by Aβo treatment for 6 h, and then by Pg stimulation for 24 h. To specially test the effect of C1q on Pg-induced inflammatory responses, microglia cells were transfected with lentivirus pLKO.1 puro containing C1qa shRNA (target sequence CCGGCTTCTATTACTTCAACTCGAG) or scramble RNA. Successful transfection was verified by analyzing C1qa mRNA and protein expression in microglia. The stably transfected microglia were then stimulated with Pg (MOI=50) for 24 h. The cells were then collected, and RNA was extracted for analyzing the gene expression of inflammatory cytokines and complement components by RT-qPCR.

### Microglia-Neuron Co-Culture

Primary neuronal cultures were prepared from the cortices of WT newborn mice using a Pierce™ primary neuron isolation kit (ThermoFisher, #88280). Neurons were used after 13-14 days of *in vitro* culturing (38). Primary microglia expressing C1qa shRNA or scramble vector were pretreated with Pg (MOI=50) for 4 h or left untreated, and then added to the neuron cultures (neuron: microglia ratio 3:1) (37). After 24 h, microglia cells were sorted from the co-cultures by FACS. The presence of synapses in microglia was analyzed by western blotting. To visualize microglia elimination of synapses, co-cultured cells were stained for IBA1 and PSD95, and then imaged on a Zeiss LSM-800 Airyscan confocal microscope as described above. Eight to ten microglia were analyzed per well, and the number of PSD95 puncta, as well as the puncta within microglia were quantified.

### Western Blotting

Equal amount of proteins (30 µg/sample) from sorted microglial lysates were run on 4%-15% mini-protean TGX stain-free precast gels (Bio-Rad) and electrotransferred to PVDF membranes (Bio-Rad). Membranes were probed with specifc antibodies against C1qa (Santa Cruz, #sc-58920), PSD95 (Santa Cruz, #sc-32290), NeuN (Abcam, #ab104224), and Tubulin (Cell signaling, #2148). Membranes were visualized using an Odyssey infrared Imaging system (LI-COR) and processed on Image J software.

### Statistical Analysis

All data are expressed as mean ± SEM and statistical significance was analyzed using GraphPad Prism (version 8.0.2, GraphPad Software Inc., San Diego, CA). One-tailed, unpaired Student *t*-tests were used for comparison between two groups. One-way ANOVA or two-way ANOVA followed by Tukey’s correction were used for analysis of more than two groups. A *P* value less than 0.05 was considered as significant.

## Results

### Pg Infection Induces Alveolar Bone Loss and Increases Complement Activation in Periodontal Tissues

Following Pg infection, significantly increased alveolar bone loss were seen at 4 months of age in WT and *App* KI mice, when compared with their respective non-infected controls (**Figures 1B, C**). In addition, increased levels of Toll-like receptor 2 (TLR2) and inflammatory cytokine (IL-1β, IL-6 and TNF-α) gene expression were detected in the gingival tissues of WT and *App* KI mice following Pg infection (**Figure 1D**). Furthermore, Pg infection significantly increased the gene expression of C1qa, the subunit A of the initiating protein C1q of the classical complement pathway, and C3, the central component of the complement system (15), in the gingival tissues of *App* KI and WT mice (**Figure 1D**). Noteworthy, without Pg infection, higher levels of inflammatory cytokine and complement gene expression, as well as alveolar bone loss, were seen in *App* KI mice than in WT mice, indicating that AD mice have a higher baseline of periodontal inflammation.

### Pg Infection Accelerates the Progression of Cognitive and Behavioral Impairment in *App* KI Mice

**Figure 2:**
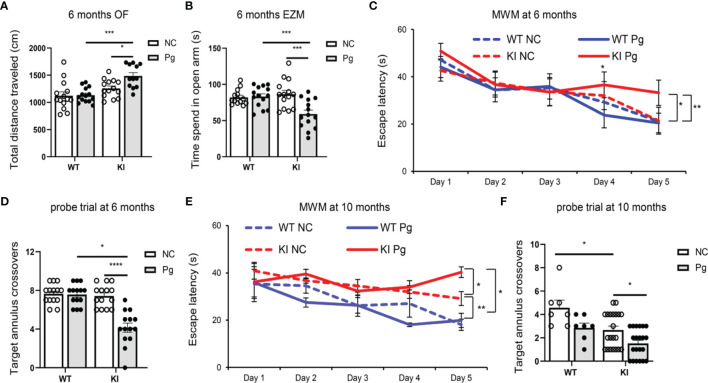
Pg infection worsens cognitive and behavior impairment in *App* KI mice. **(A)** Total distance traveled in open field (OF) tests at 6 months of age. cm, centimeter. **(B)** Time spent in open arm in elevated zero maze (EZM) tests at 6 months of age. s, second. **(C, E)** Escape latency in Morris water maze (MWM) tests at 6 **(C)** and 10 **(E)** months of age. **(D, F)** Target annulus crossovers in probe trial tests at 6 **(D)** and 10 **(F)** months of age. A-D, n=14/group; E-F, n=21/group (*App* KI mice), n=7/group (WT mice). Data are expressed as mean ± SEM. *P < 0.05, **P < 0.01, ***P < 0.001, ****P < 0.0001 by two-way ANOVA followed with Tukey correction.

The main clinical symptom of AD is a decline in memory and cognitive function (39). Although associations between periodontitis and AD have been made, conclusive evidence of the influence of periodontitis on AD has not been clearly established. To assess the impact of periodontitis on the development of cognitive and behavioral impairment, behavioral tests were carried out at three time points (**Figure 1A**). We did not observe any significant behavior changes between any of the groups at 4 months of age (data not shown). However, at 6 months of age, compared to non-infected *App* KI mice, Pg-infected *App* KI mice showed significantly increased locomotor activity by OF test (**Figure 2A**), increased anxiety-related behavior by EZM test (**Figure 2B**), and impaired spatial learning and memory by MWW test and probe trial (**Figures 2C, D**). No significant difference was observed in WT mice with or without Pg infection, or between non-infected WT and *App* KI mice. At 10 months of age, non-infected *App* KI mice developed clear deficits in the MWM test when compared with non-infected WT mice (**Figures 2E, F**). In addition, Pg infection significantly increased the cognitive deficits in *App* KI mice. These results demonstrate that periodontal infection accelerates the progression of cognition and behavioral impairment in AD mice.

### Pg Infection Exacerbates Brain Aβ Production and Amyloid Plaque Deposition in AD Mice

**Figure 3:**
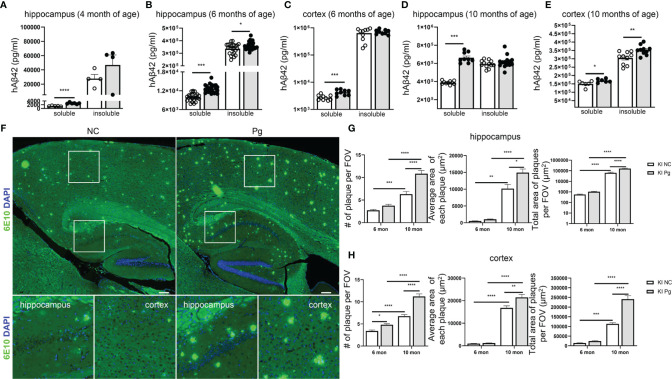
Pg infection accelerates Aβ production and plaque formation in *App* KI mice. **(A–E)** Levels of soluble and insoluble hAβ42 in the brains of control and Pg-infected *App* KI mice at 4, 6 and 10 months of age. A, n=4-6/group; B, n=24/group; C-E, n=6-10/group. Samples were run in duplicate in ELISA. **(F)** Representative micrographs showing Aβ plaques formation in *App* KI mice at 10 months of age. Whole brain images were stitched with 30 series micrographs captured at 10 x objective using a KEYENCE BZ-800 microscope. Boxed areas were further enlarged. Aβ plaques were labeled with 6E10 (green) and cell nuclei were labeled with DAPI (blue). **(G, H)** Quantification of the number of Aβ plaques, average plaque size, and total plaque-occupied area per field of view (FOV) in the hippocampus **(G)** and cortex **(H)** of *App* KI mice at 6 and 10 months of age. Five representative regions of the cortex and hippocampus were captured at 40x objective from each brain section and quantified (n=5-7/group). Data are expressed as mean ± SEM. *P < 0.05, **P < 0.01, ***P < 0.001, ****P < 0.0001 by unpaired Student *t*-test **(A–E)**, or by two-way ANOVA followed with Tukey correction **(G, H)**.

The development of amyloid plaque, composed mainly of the peptide Aβ, is a pathological hallmark of AD (40). We next questioned how Pg infection influences brain Aβ accumulation and amyloid plaque deposition in *App* KI mice. At 4 months of age, accumulation of soluble and insoluble hAβ42 peptide was noted in the brains of *App* KI mice, and Pg infection significantly increased the accumulation of soluble hAβ42 in the hippocampus of *App* KI mice (**Figure 3A**). Time-dependent accumulation of hAβ42 in the brains of non-infected *App* KI mice was seen at 6 and 10 months of age (**Figures 3B–E**), compared to the level at 4 months. In addition, Pg infection further enhanced hAβ accumulation in *App* KI mice at 6 and 10 months of age. Consistently, immunohistochemical analysis of brain sections demonstrated significantly increased amyloid plaque deposition in non-infected *App* KI mice at 10 months of age compared to 6 months of age, as measured by the number of plaques, the average size of plaques, and the total area occupied by the plaques in hippocampus and cortex (**Figures 3F–H**). Furthermore, Pg infection significantly increased amyloid plaque deposition in *App* KI mice at 6 and 10 months of age.

### Pg Invades the Brain and Potentiates Neuroinflammation and Complement Activation in AD Mice

**Figure 4:**
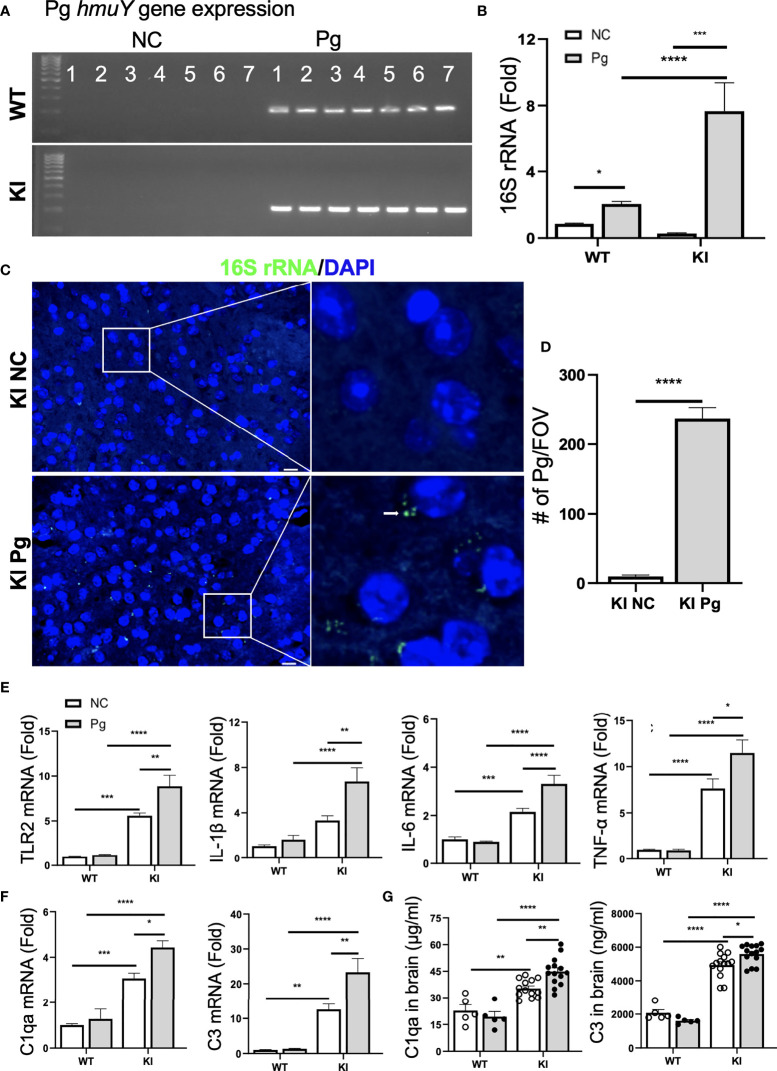
Pg invades the brain and induces neuroinflammation and complement activation. **(A)** Pg-specific *h*muY gene expression in brains from WT and *App* KI mice. **(B)** Pg-specific 16S rRNA expression in brains from WT and *App* KI mice. The levels of gene expression were normalized to GAPDH and shown as fold changes. Samples were run in duplicates (n=7/group). **(C)** Representative micrographs depicting intracellular localization of Pg in the cortex of brains from *App* KI mice. Pg were probed with 16S rRNA with FITC (green, arrowhead) and nuclei were labeled with DAPI (blue). Boxed areas were further enlarged. Scale bar, 10 µm. **(D)** Quantification of Pg in brain sections from non-infected and Pg-infected *App* KI mice. Five representative regions were captured at 40 x objective from each brain section and quantified (n=5-7/group); FOV, field of view. **(E)** The relative gene expression of inflammatory cytokines and TLR2 in brain tissues from WT and *App* KI mice. Samples were done in duplicates (n=5-7/group). **(F)** The relative gene expression of C1qa and C3 in brain tissues from WT and *App* KI mice. Samples were run in duplicates (n=5-7/group). **(G)** The levels of C1qa and C3 in the brains of WT and *App* KI mice. Samples were run in duplicates (WT mice, n=5-7/group; *App* KI mice, n=10-12/group). Data are expressed as mean ± SEM; *P < 0.05, **P < 0.01, ***P < 0.001, ****P < 0.0001 by two-way ANOVA followed with Tukey correction **(B, E–G)** or by unpaired Student *t*-test **(D)**.

Recent studies have shown that the number of Pg, and its LPS and gingipains, were significantly higher in the autopsied brain tissues of AD patients than in non-demented controls (10, 11), suggesting that Pg can pass the blood-brain barrier (BBB). To verify Pg brain invasion following oral gavage infection, we first assessed the presence of the *hmuY* gene, a highly specific gene essential for Pg survival and virulence (33), in the brains of WT and *App* KI mice. While non-infected WT and *App* KI controls were negative for the *hmuY* gene, both Pg-infected WT and *App* KI brains were positive for this gene (**Figure 4A**). We next analyzed the presence of Pg 16S rRNA. Both WT and *App* KI brains showed significantly increased expression of Pg 16S rRNA following Pg infection (**Figure 4B**). Noteworthy, a significantly higher level of Pg 16S rRNA was detected in the brain tissues from *App* KI mice as compared to those from WT mice following Pg infection. The presence of Pg in the brain tissues of Pg-infected *App* KI mice was further ascertained by FISH using Pg specific 16S rRNA probe. Hybridization of the 16S rRNA probe to bacterial aggregates was located at the perinuclear regions of brain cells in Pg-infected *App* KI mice (**Figures 4C, D**). No Pg 16S rRNA was detected in non-infected *App* KI mice brains. These results demonstrate that upon the establishment of periodontal infection, Pg can access the brain and that AD mice are more susceptible to Pg brain invasion than WT mice.

Neuroinflammation is a critical component of neurodegenerative diseases like AD (41). Thus, we next determined how oral Pg infection affected the neuroinflammatory status of experimental mice. Without Pg infection, *App* KI mice showed significantly higher levels of TLR2 and inflammatory cytokine gene expression than WT mice (**Figure 4E**). Pg infection further enhanced the expression of these genes in the brains of *App* KI mice, while a minimal inflammatory response was observed in WT mice following Pg infection. Similar results were seen with C1qa and C3 gene and protein expression in the brains of WT and *App* KI mice. Non-infected *App* KI mice showed significantly higher levels of brain C1qa and C3 gene and protein expression than WT mice, and Pg infection further enhanced their expression in the brains of *App* KI mice (**Figures 4F, G**). However, minimal C1qa and C3 response was noted in the brains of WT mice following Pg infection. These results suggest that periodontal infection contribute significantly to the neuroinflammatory milieu established by AD possibly *via* activation of complement system.

### Pg Infection Enhances Microglia Activation and Their Colocalization With C1q in *App* KI Mice

**Figure 5:**
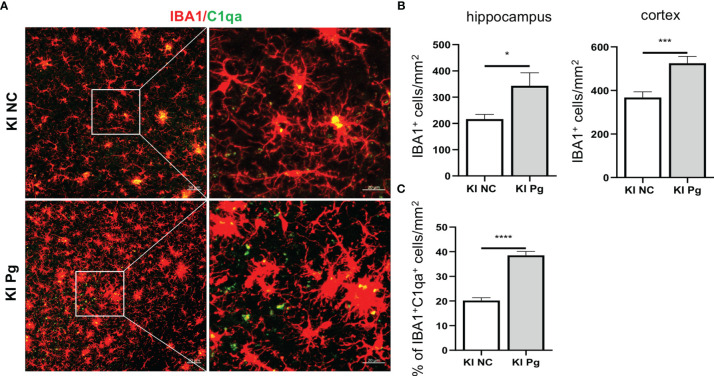
Pg activates microglia in *App* KI mice and co-localize with complement C1q. Brain sections from 6-month-old non-infected and Pg-infected *App* KI mice were immune-stained with antibodies against IBA1and C1qa. **(A)** Representative Z-stack images of brain sections depicting the spatial association between microglia (red) and C1qa (green). **(B)** Quantification of IBA1^+^ cells in the hippocampus and cortex regions. n=6-7 mice/group. **(C)** Percentage of IBA1^+^C1qa^+^ microglia in non-infected and Pg-infected *App* KI mice. Five representative micrographs of the cortex and hippocampus regions from each mouse were analyzed (n=5-7 mice/group). Data are expressed as mean ± SEM. *P < 0.05, ***P < 0.001, ****P < 0.0001 by unpaired Students *t*-test.

As the major immune cells in the CNS, microglia activation is an invariable feature of AD pathology, and activated microglia represent a major source of inflammatory factors in AD (42). We next assessed the activation of microglia and the spatial association between microglia and C1q in *App* KI mice. Pg infection significantly increased the number of IBA1^+^ microglia in *App* KI mice (**Figures 5A–C**). In addition, a significant increase in the number of C1qa^+^IBA1^+^ cells was observed in Pg-infected *App* KI mice.

### Pg Amplifies Aβ-Primed Microglia Activation *via* C1q

**Figure 6:**
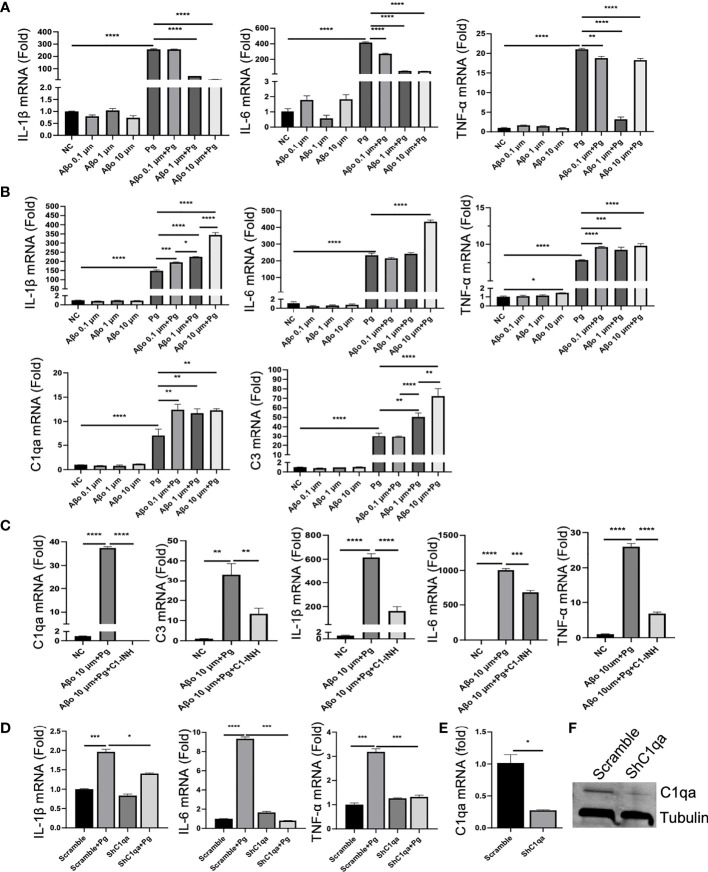
Effect of Aβo and complement activation on Pg-induced microglial inflammatory responses. **(A, B)** Aβo on Pg-induced cytokine production and complement activation. Primary microglia were treated with Aβo (0.1, 1 or 10 µM), Pg (MOI=50), or co-stimulated with Aβo and Pg for 24 h **(A)**, or pretreated with Aβo for 6 h followed by Pg stimulation for 24 h **(B)**. Relative gene expression of IL-1β, IL-6, TNF-α, C1qa, and C3 was analyzed by RT-qPCR. **(C)** C1 inhibition on Pg-induced inflammatory gene expression by microglia. Primary microglia were untreated or pretreated with C1-INH for 4 h, and then treated with Aβo (10 µM) for 6 h followed by Pg (MOI=50) for 24 h. Relative gene expression of C1qa, C3, IL-1β, IL-6, and TNF-α in microglia were analyzed by RT-PCR**. (D)** Effect of C1qa depletion on Pg-induced cytokine gene expression by microglia. Primary microglia cells expressing scramble or C1qa shRNA vectors were treated with Pg (MOI=50) for 24 h. Relative gene expression of IL-1β, IL-6, TNF-α in microglia were analyzed by RT-PCR. Expression of C1qa mRNA **(E)** and protein **(F)** in primary microglia verified by RT-PCR and Western blot. Samples were run in duplicates. Data are expressed as mean ± SEM of three independent experiments; *P < 0.05, **P < 0.01, ***P < 0.001, ****P < 0.0001 by one-way ANOVA followed with Tukey correction.

Given that AD mice exhibited neuroinflammation without Pg infection, and that Pg entered the brains of WT mice but induced minimal neuroinflammation compared to AD mice, we questioned if a potential interplay between Pg and Aβ could result in the increased neuroinflammation observed in AD mice. Primary microglia were cultured with different concentrations of Aβo, Pg, Aβo together with Pg, or pre-treated with Aβo followed by Pg infection. Interestingly, while Aβo alone at various doses induced very low levels of inflammatory cytokine and complement gene expression in microglia (**Figures 6A, B**), co-stimulation of microglia with Aβo and Pg at the same time decreased the gene expression of inflammatory cytokines as compared to Pg stimulation alone (**Figure 6A**). However, pre-treatment of microglia with Aβo significantly potentiated Pg-induced inflammatory cytokine and complement gene expression (**Figure 6B**).

To assess if the increased inflammatory response by Pg in Aβ-primed microglia was mediated by complement activation, microglia were pre-treated with C1-INH, a protease inhibitor of the classical complement component C1 (43), followed by Aβo and then Pg stimulation. Our results showed that C1-INH significantly decreased Pg-induced complement activation and cytokine production in Aβ-primed microglia (**Figures 6C, D**). To further verify if C1q spurs neuroinflammation in the presence of Pg, primary microglia were transfected with shC1qa or scramble vectors and then treated with Pg. Knock-down of C1qa significantly reduced Pg-induced inflammatory cytokine gene expression in microglia (**Figures 6D–F**). These results suggest that C1q plays a critical role in the amplification of microglia activation and neuroinflammation in AD mice following Pg infection.

### Pg Infection Exacerbates Synapse Loss and the Colocalization of C1q With Synaptic Puncta in *App* KI Mice

**Figure 7:**
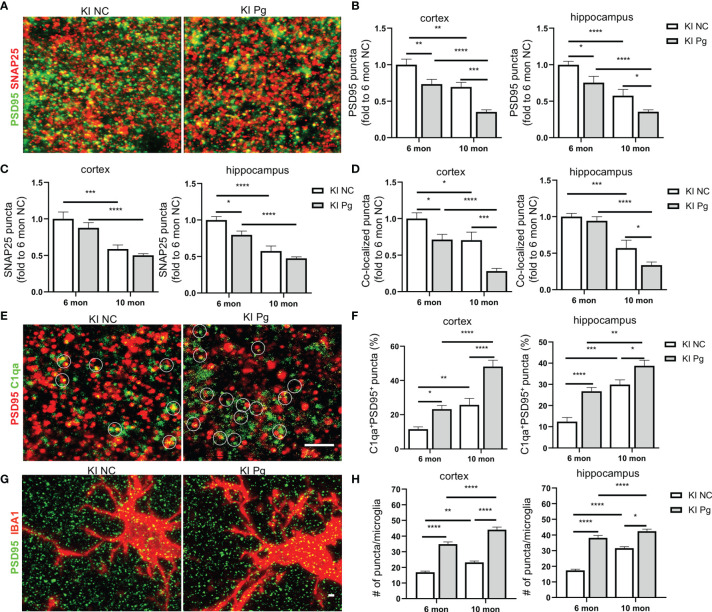
Pg infection enhances microglial elimination of synapses in *App* KI mice. **(A)** Representative high magnification Z-stack images showing SNAP 25 (presynaptic marker, red) and PSD95 (postsynaptic marker, green) synaptic terminals in the cortex and hippocampus from non-infected and Pg-infected *App* KI mice at 10 months of age. **(B–D)** Quantification of PSD95 puncta **(B)**, SNAP25 puncta **(C)**, and the co-localized PSD95 and SNAP25 puncta **(D)** in the cortex and hippocampus CA1 regions from non-infected and Pg-infected *App* KI mice at 6 and 10 months of age. Scale bar: 2 µm. **(E)** Representative high magnification Z-stack images of C1qa (green) and PSD95 (red) co-stained puncta in the cortex and hippocampus of the brains from non-infected and Pg-infected *App* KI mice at 10 months of age. Circles show examples of C1qa puncta co-localized with PSD95 puncta. Scale bar: 4 µm. **(F)** Quantification of co-stained C1qa and PSD95 in the cortex and hippocampus CA1 regions from non-infected and Pg-infected *App* KI mice at 6 and 10 months of age. **(G)** Representative high magnification Z-stack images of subicular microglia (IBA1^+^, red) co-stained with PSD95 (green) from non-infected and Pg-infected *App* KI mice at the 10 months of age, displaying elimination of synapses by microglia. Scale bar, 5 µm. **(H)** Quantification of engulfed PSD95 puncta density in microglia in the cortex and hippocampus CA1 regions of the brains from non-infected and Pg-infected *App* KI mice at 6 and 10 months of age. Data are expressed as mean ± SEM (n=4-7 mice/group). *P < 0.05, **P < 0.01, ***P < 0.001, ****P < 0.0001 by two-way ANOVA followed with Tukey correction.

One of the prominent pathological features of AD is the early loss of synapses (44). In addition, studies have shown that progressive brain C1q accumulation is associated with cognitive decline and memory impairment in aging, *via* microglia engulfment of the C1q-tagged synapses (45). Given the significantly increased brain C1q expression and remarkable memory deficits in *App* KI mice following Pg infection, we evaluated if synapses were altered as a result of periodontal infection, and its correlation with C1q expression. A significant loss of pre- and postsynaptic puncta (marked by SNAP25 and PSD95, respectively), as well as the colocalization of pre- and postsynaptic puncta, in the hippocampus and cortex of non-infected *App* KI mice were seen at 10 months of age compared to 6 months of age (**Figures 7A–D**). In addition, Pg infection significantly increased the loss of synaptic puncta in *App* KI mice at 6 and 10 months of age than their respective non-infected controls. Furthermore, a significant increase in the numbers of C1qa-labeled PSD95 puncta in the hippocampus and cortex was observed in non-infected and Pg-infected *App* KI mice at 10 months of age compared to that at 6 months of age, and Pg-infected *App* KI mice showed a significantly higher number of C1qa^+^PSD95^+^ puncta than their non-infected controls at 6 months and 10 months of age (**Figures 7E, F**). Moreover, significant increase numbers of PSD95 puncta within microglial cell bodies was observed in the cortex and hippocampus of *App* KI mice at 10 months of age compared to those at 6 months of age, and Pg infection significantly increased the numbers at 6 and 10 months of age (**Figures 7G, H**). These results suggest that C1q and microglia communicate to regulate synapse pruning, and that periodontal infection potentiates C1q-mediated synapse engulfment by microglia.

### Blocking C1qa Reduces Pg Induced Microglia-Mediated Synapse Loss *In Vitro*

**Figure 8:**
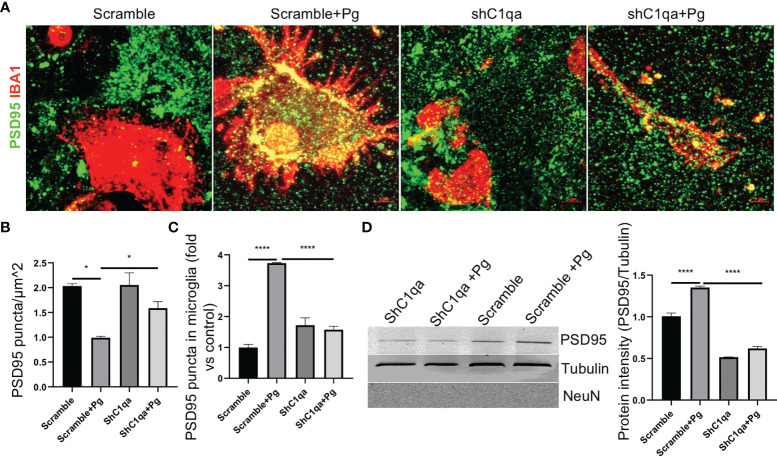
Depletion of C1qa prevents Pg-induced synapse loss by microglia *in vitro*. **(A)** Representative Z-stack images of neuro-microglia (expressing shC1q or scramble vector) co-cultures in the presence or absence of Pg (MOI=50). Cultures were stained with IBA1 (red) and PSD95 (green). Scale bar: 5 µm. **(B, C)** Quantification of PSD95 puncta in the co-cultures **(B)** and in microglial cell bodies **(C)**. **(D)** Western blot analysis of PSD95 puncta in FACS-sorted IBA1^+^ microglia lysates from the co-cultures. NeuN, neuronal marker. Densitometric analysis was performed using ImageJ software, and normalized to tubulin and expressed as fold changes over scramble control. Data are expressed as mean ± SEM of three independent experiments. *P < 0.05, ****P < 0.0001 by one-way ANOVA followed with Tukey correction.

To further confirm if the increased synapse removal by microglia in the presence of Pg was C1q dependent, primary microglia expressing C1qa shRNA or scramble vectors were co-cultured with primary neurons in the presence or absence of Pg, and the expression of PSD95 puncta in the co-cultures were analyzed. With scramble shRNA expression, PSD95 density in the co-cultures was reduced by ~50% following Pg stimulation (**Figures 8A, B**), demonstrating that Pg infection enhances the loss of synapses. However, with shC1qa expression, no significant loss of PSD95 was seen following Pg stimulation. Importantly, the number of PSD95 puncta in Pg-treated co-cultures with shC1qa expression was significantly higher than that in Pg-treated co-cultures with scramble shRNA expression, indicating that depletion of C1qa in microglia prevents Pg-induced synapse loss. We next verified the presence of PSD95 puncta within microglia in the co-cultures. Increased number of PSD95 puncta were observed within microglia expressing scramble vectors after Pg stimulation (**Figures 8A, C**). However, no increase in PSD95 puncta within microglia expressing shC1qa was seen following Pg stimulation. To further confirm these results, microglia cells were sorted out from the co-cultures by FACS. Pg infection led to an increased level of PSD95 expression in the microglial cell lysates from the co-cultures with scramble shRNA expression in microglia (**Figure 8D**). However, no increase in PSD95 expression was seen in the microglial cell lysates from the co-cultures with shC1qa expression. No NeuN (neuronal cell marker) signal was detected in any of the microglial cell lysates, indicating that there was no contamination of neurons in the sorted microglia and that the observed PSD95 intensity was due to microglial engulfment of neuronal synapses. Taken together, these results indicate that C1q expression in microglia is necessary for Pg-induced enhancement of synapse engulfment by microglia.

## Discussion

Increasing evidence suggests that periodontitis is associated with and may contributes to the development of AD. However, the mechanisms underlying such association have not yet been delineated. In the present study, we provide new evidence that the neuroinflamamtory staus established by AD, is greatly complicated by periodontal infection and the consequential entry of Pg into the brain *via* Aβ-primed microglial activation, thus unquestionably and significantly aggravating AD, and that Pg-induced brain overactivation of complement C1q is critical for periodontitis-associated acceleration of AD progression by amplifying microglial activation, neuroinflammation, and tagging synapses for microglial engulfment.

Periodontitis-related bacteremia, as well as the migration of Pg from gingival tissues to distant tissues *via* intravascular dissemination, has been reported in animal and human studies (46, 47). In addition, periodontitis may induce a systemic inflammatory state through mechanisms that include dissemination of pro-inflammatory cytokines (6). In this study, we were able to detect the presence of Pg in the brains of WT and AD mice following Pg infection. Importantly, a significantly higher amount of Pg were detected in the brains of AD mice than WT mice. One reason for the increased vulnerability of AD mice to Pg invasion of the brain may due to the increased BBB permeability related to increased activation of the innate immune response in the brains of AD mice, thus allowing for easier translocation of pathogens through the barrier (48). Along this line, we observed a pre-existing pro-inflammatory cytokine response in the brains of AD mice without Pg infection, and Pg infection further enhanced the neuroinflammation in AD mice.

Microglia are belived to be the main source of C1q in the brain (49), consistent with this notion, we found that Pg infection significantly increased the colocalization of C1q with microglia, and C1q plays an important role in Pg-induced inflammation in microglia. Previous studies have reported that the complement system is hyper-activated in the brain tissues from AD patients and mouse models (18, 19), inhibition or lack of complement components could ameliorate AD-relevant characters in multiple mouse models of AD (18, 50, 51). In addition, absence of C1q leads to less neuropathology in AD mice. On the other hand, activated complement fragments were abundant in the gingival crevicular fluids (GCF) and the chronically inflamed gingiva from patients with periodontitis, but were either undetectable or present in very low levels in the GCF and gingiva of healthy control individuals (52). Periodontal treatment that decreased clinical indices of periodontal inflammation has also been shown to decrease complement activity in GCF (53). Moreover, interception of the complement cascade protected mice and non-human primates from periodontitis (54). Thus, a dysregulated complement system may be a driver of chronic, non-resolving neuroinflammation in AD in the presence of periodontal infection.

Microglia are long-lived tissue-resident macrophages in the CNS (55). Like macorphages, microglia express pattern recognition receptors, e.g., TLRs, and can be activated by multiple exogeous TLR ligands (56). In addition, microglia are believed to possesse innate immune memeory after experiencing a primary “priming” or “desensitizing” stimulus, react with a stronger (immune training) or weaker (immune tolerance) immune response to a subsequent stimulus (57, 58). Pg mainly signals through TLR2 to induce periodontal inflammation and bone loss (59). Interestingly, TLR2 is also a primary receptor for Aβ peptides to trigger neuroinflammation (60). Thus, Aβ accumulation in the brain may underline the pre-existed brain TLR2 activation and neuroinflammation in AD mice in the absence of Pg infection. Interestingly, our studies showed that Aβο alone was unable to activate mciroglial cells *in vitro*. Yet, pretreatment of microglia cells with Aβo followed by Pg stimulation led to a significant increase in Pg-induced inflammatory response even when a very low amount of Aβo was used, which is consistent with our *in vivo* findings that Pg infection amplified compelement activation and neuroinflamamtion in AD mice. Therefore, it is likely that Aβ accumulation in AD mice may serve as a first “hit” to prime or train microglia, while Pg brain invasion as a second “hit” to activate the primed microglia, leading to exaggerated neuroinflamamtion in AD in the presence of periodontal infection. It is also possible that brain dissemination of Pg or inflammatory mediators can prime microglia for subsequent Aβ or inflammatory stimuation (61), which would suggest that periodontitis not only aggravates the severity of AD, but also increases the host susceptibility to AD.

It is important to point out that, in our study, minimal brain complement activation and neuroinflammation were seen in WT mice following Pg infection, despite Pg brain invasion. A possible explanation is that Aβ production in a healthy brain may help sequester the invading pathogens. In this regard, previous studies have shown that Aβ can protect culture cells from microbial infection by forming fibrils that entrap the pathogens and destroy cell membranes (62). In line with these observations, we found that *in vitro* co-stimulation of microglial cells with Aβo and Pg at the same time significantly impaired Pg-induced cytokine production.

Emerging research indicates that early and extra synapse loss is a predictor of cognitive impairment and the degree of synapse loss correlates most strongly with cognitive decline in AD (63). Several lines of evidence support the notion that synapse loss in AD is caused by complement-mediated synaptic pruning by microglia, and that C1q deficiency leads to less synapse loss and neuropathology in AD mice (18, 51, 64). Therefore, we reasoned that elevated complement and microglia activation induced by Pg infection would exacerbate synapse loss in AD mice. Indeed, we observed a significant loss of synapses and increased co-localization of C1q with synapses in AD mice following Pg infection. In addition, more synaptic puncta were observed within microglia following Pg infection, and this engulfment was prevented by depleting C1qa. However, how C1q-labeled synapses provide an “eat me” signal to microglia needs to be addressed in future studies. Furthermore, it will also be of interest to see if C1q inhibition or deficiency *in vivo* is sufficient to mitigate Pg-mediated acceleration of AD pathology.

**Figure 9:**
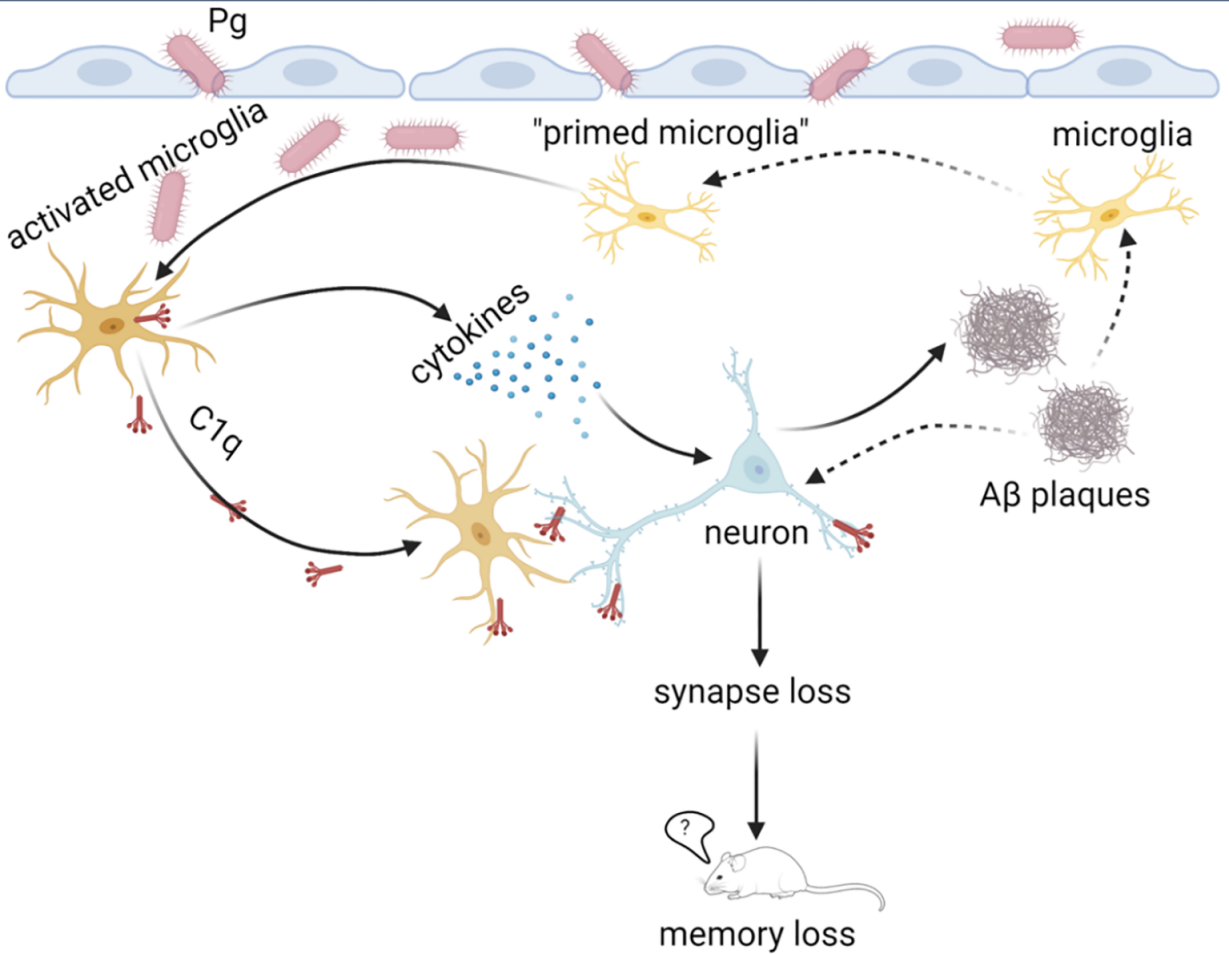
Proposed “two-hit” model of AD progression in the presence of periodontitis. The accumulation of Aβ in the brain with AD may serve as the first “hit” to prime microglia and induce low levels of complement activation and neuroinflammation. In the presence of periodontitis, periodontal pathogens may invade the brain and serve as the second “hit” to amplify neuroinflammation of the Aβ-primed microglia and facilitate synapse loss. Complement C1q is critical for Pg-mediated acceleration of AD progression by tagging synapses for microglia engulfment. This image was created by Biorender.com.

In conclusion, we propose a “two-hit” model of periodontitis-associated aggravation of AD progression, with brain amyloid β accumulation as the first “hit”, and Pg invasion of the brain as the second “hit” to facilitate microglial overactivation and synapse loss in AD in the presence of periodontitis (**Figure 9**). Furthermore, periodontitis-induced overactivation of complement C1q is critical in aggravating microglial priming and activation, leading to an exacerbated neurodegeneration in AD. Thus, simply reducing Aβ burden alone without eliminating periodontal infection may not completely break a vicious cycle formed by Aβ deposition, complement activation, and neuroinflammation in AD in the presence of periodontitis. Future studies will determine the regulation of microglia priming and if targeting certain complement components could ameliorate periodontitis and thus AD progression in the presence of periodontal infection. Knowledge obtained from these studies will fill the gap in our understanding of the association between periodontitis and AD, and will pave the way for targeting microbial etiology to ameliorate the clinical manifestations of AD and lower AD prevalence.

## Data Availability Statement

The original contributions presented in the study are included in the article/**Supplementary Material**. Further inquiries can be directed to the corresponding author.

## Ethics Statement

The animal study was reviewed and approved by University of Alabama at Birmingham Institutional Animal Care and Use Committee.

## Author Contributions

PZ conceptualized and supervised the study. XH planned and performed the experiments, and analyzed data. ZL assisted with the animal experiments and WL assisted with imaging processing and data analysis. PZ and XH wrote the manuscript. JK, SM, SB, LP-M, QW, and ER provided expertise and resources, and critically reviewed the manuscript. TS and TCS provided the *App* KI mice. All authors read and approved the final manuscript.

## Funding

This work was supported by National Institute of Dental and Craniofacial Research (NIDCR) grant DE026465 (to PZ). ZL was supported by a NIDCR training grant R90 DE023056. UAB Comprehensive Flow Cytometry Core is supported by NIH P30 AR048311 and P30 AI27667. UAB Behavior Assessment Core is supported by NIH P30 NS47466. Confocal images were taken at the UAB Comprehensive Neuroscience Center and the UAB Civitan International Research Center.

## Conflict of Interest

SB is the CSO & Co-Founder of CNine Biosolutions, LLC.

The remaining authors declare that the research was conducted in the absence of any commercial or financial relationships that could be construed as a potential conflict of interest.

## Publisher’s Note

All claims expressed in this article are solely those of the authors and do not necessarily represent those of their affiliated organizations, or those of the publisher, the editors and the reviewers. Any product that may be evaluated in this article, or claim that may be made by its manufacturer, is not guaranteed or endorsed by the publisher.
