# Supplementary material for: Periodontal Infection Aggravates C1q-Mediated Microglial Activation and Synapse Pruning in Alzheimer’s Mice
Source: Front Immunol. 2022 Feb 1;13:816640. doi: 10.3389/fimmu.2022.816640 (PMC8845011; doi:10.3389/fimmu.2022.816640)
Supplement: Supplementary file 1 [file Table_1.docx]

**Table S1. Sequences of primers used in this study**

|  | Forward primer (5ʹ to 3ʹ) | Reverse primer (5ʹ to 3ʹ) |
| --- | --- | --- |
| C1qa | AAAGGCAATCCAGGCAATATCA | TGGTTCTGGTATGGACTCTCC |
| C3 | ACTGTGGACAACAACCTACTGC | GCATGTTCGTAAAAGGCTCGG |
| GAPDH | AGGTCGGTGTGAACGGATTTG | TGTAGACCATGTAGTTGAGGTCA |
| *hmu*Y | GCTTCGAAATACGAAACGTG | TATATCCGTCTGTCGGAACG |
| IL-1β | ACAGAATATCAACCAACAAGTGATATTC | GATTCTTTCCTTTGAGGCCCA |
| IL-6 | TAGTCCTTCCTACCCCAATTTCC | TTGGTCCTTAGCCACTCCTTC |
| 16S rRNA | CTTGACTTCAGTGGCGGCA | AGGGAAGACGGTTTTCACCA |
| TNF-α | CCCTCACACTCAGATCATCTTCT | GCTACGACGTGGGCTACAG |
| TLR2 | CCTGTTGATCTTGCTCGTAGGTGCC | CTACCTGGAGTGGCCCTTGGATGAA |
